# Evaluation of diagnostic efficacy for localization of parathyroid adenoma in patients with primary hyperparathyroidism undergoing repeat surgery

**DOI:** 10.1007/s00423-021-02191-z

**Published:** 2021-05-16

**Authors:** Anne Hendricks, Christina Lenschow, Matthias Kroiss, Andreas Buck, Ralph Kickuth, Christoph-Thomas Germer, Nicolas Schlegel

**Affiliations:** 1 Department of General, Visceral, Vascular and Pediatric Surgery, University Hospital Würzburg, Oberduerrbacherstrasse 6, 97080 Wuerzburg, Germany; 2 Department for Diabetology and Endocrinology, Clinic for Internal Medicine I, University Hospital Wuerzburg, Oberduerrbacherstrasse 6, 97080 Wuerzburg, Germany; 3 Clinic for Nuclear Medicine, University Hospital Wuerzburg, Oberduerrbacherstrasse 6, 97080 Wuerzburg, Germany; 4 Institute for Diagnostic and Interventional Radiology, University Hospital Wuerzburg, Oberduerrbacherstrasse 6, 97080 Wuerzburg, Germany

**Keywords:** Primary hyperparathyroidism (pHPT), Repeat surgery, Diagnostics, Imaging, Preoperative localization

## Abstract

**Purpose:**

Repeat surgery in patients with primary hyperparathyroidism (pHPT) is associated with an increased risk of complications and failure. This stresses the need for optimized strategies to accurately localize a parathyroid adenoma before repeat surgery is performed. However, evidence on the extent of required diagnostics for a structured approach is sparse.

**Methods:**

A retrospective single-center evaluation of 28 patients with an indication for surgery due to pHPT and previous thyroid or parathyroid surgery was performed. Diagnostic workup, surgical approach, and outcome in terms of complications and successful removement of parathyroid adenoma with biochemical cure were evaluated.

**Results:**

Neck ultrasound, sestamibi scintigraphy, C11-methionine PET-CT, and selective parathyroid hormone venous sampling, but not MRI imaging, effectively detected the presence of a parathyroid adenoma with high positive predictive values. Biochemical cure was revealed by normalization of calcium and parathormone levels 24–48h after surgery and was achieved in 26/28 patients (92.9%) with an overall low rate of complications. Concordant localization by at least two diagnostic modalities enabled focused surgery with success rates of 100%, whereas inconclusive localization significantly increased the rate of bilateral explorations and significantly reduced the rate of biochemical cure to 80%.

**Conclusion:**

These findings suggest that two concordant diagnostic modalities are sufficient to accurately localize parathyroid adenoma before repeat surgery for pHPT. In cases of poor localization, extended diagnostic procedures are warranted to enhance surgical success rates. We suggest an algorithm for better orientation when repeat surgery is intended in patients with pHPT.

## Introduction

Primary hyperparathyroidism (pHPT) is the third most common endocrine disorder [1]. It is diagnosed biochemically and is defined as an inappropriately high level of total serum calcium due to maladjusted, elevated levels of parathormone (PTH). In 70–80% of cases, a single solitary parathyroid adenoma is causative for pHPT, while 20–30% of cases are accounted for by multiglandular disease. On the other hand, parathyroid carcinomas are relatively rare [2–4].

The therapy of choice and only curative treatment for pHPT is surgical resection of the responsible adenoma or, in cases of multiglandular disease, total or subtotal parathyroidectomy of all glands with reimplantation of parathyroid tissue. Therapeutic success is characterized by the normalization of calcium and PTH levels [1].

Although surgery is remarkably effective, with success rates of up to 96%, persistent or recurrent pHPT is diagnosed in 1 to 6% of all pHPT patients [5, 6]. Persistent pHPT is defined as hypercalcemia within 6 months of surgery. In cases of recurrent pHPT, hypercalcemia recurs at least 6 months after surgery although it is frequently diagnosed much later. After the diagnosis of persistent or recurrent pHPT, re-operation is usually performed [7]. Additionally, many patients with a history of thyroid surgery require repeat surgery when pHPT is diagnosed. In this cohort of patients, it is often not known whether all (unaffected) parathyroid glands were preserved during the primary operation [8].

Irrespective of patient background, repeat operations are associated with a number of problems including incomplete surgical history, scar formation, adhesions, and altered anatomy, which all hinder the correct and immediate localization of an adenoma of the parathyroid gland and can result in complications and increased morbidity. This also applies to previous surgery of the thyroid gland [8]. The major procedure-specific complications are recurrent laryngeal nerve (RLN) palsy and hypoparathyroidism. Transient recurrent laryngeal nerve (RLN) palsy occurs in about 11% of all cases, whereas repeated surgery causes permanent RLN palsy in 3–9% of cases. In contrast, only 0.3–0.6% of patients experience this complication after primary surgery [9]. Furthermore, transient postoperative hypocalcemia is detected in up to 81% and permanent hypoparathyroidism in up to 13% of the cases receiving repeated surgery, while the risk is again significantly lower in primary surgery (7.8%) [2, 9–12]. However, the cure rates of persistent and recurrent pHPT, at least in specialized centers, range from 93 to 97%, which is almost identical to primary surgery [8, 9, 11].

With these outcomes in mind, the importance of preoperative localization studies has to be stressed as they improve the cure rate before re-operation [13, 14]. Furthermore, they reduce the extent of surgical exploration and are appropriate to detect ectopic parathyroid adenomas, which are more prevalent in re-explored patients. Presently, a number of noninvasive and invasive imaging modalities are available for use before re-operation in recurrent or persistent pHPT with varying sensitivities and predictive values. While for many years two concordant localization studies have generally been recommended before repeat surgery, data supporting this recommendation are missing. Moreover, even if imaging successfully detects a potential adenoma, the risk of false-positive results depending on the imaging modality remains, which may mislead the surgeon. Therefore, from a surgical point of view, it is unclear how much preoperative diagnostic is required and justified to increase the probability of a successful procedure.

This prompted us to evaluate the success rate and diagnostic workup of the cohort of patients at our center with repeat surgery for pHPT after previous thyroid or parathyroid surgery, respectively. We compared the results with the current literature to present a constructive, applicable, and economic algorithm for imaging before re-operation in cases of persistent or recurrent pHPT, or after thyroid surgery.

## Patients and methods

### Setting and study design

A retrospective single-center analysis of all patients that underwent repeat neck surgery because of primary hyperparathyroidism was performed. Inclusion criteria were age ≥18 years, diagnosis of primary hyperparathyroidism, and a history of previous thyroid and/or parathyroid surgery. The designation “thyroid surgery” includes every surgical procedure affecting the thyroid gland such as thyroidectomy, hemithyroidectomy, or subtotal thyroid resection.

All patients with pHPT and thyroid and/or parathyroid surgery between January 1st, 2006, and October 15th, 2020, were extracted from the local database after ethical approval (number 20190205 01). Thereafter, a retrospective analysis of all patients’ parameters was performed.

### Data acquisition

Clinical data (patient characteristics: age, gender; primary surgery: parathyroid surgery with localization of the parathyroid adenoma or thyroid surgery; serum chemistry: parathormone (PTH), calcium; vocal cord mobility, endocrine workup, imaging studies and results; surgery: procedure, time, intraoperative PTH values; histopathological findings; complications: bleeding, hypocalcemia, paresthesia, recurrent laryngeal nerve palsy; postoperative parameters: serum calcium, PTH) of patients with repeat surgery for pHPT were retrieved from the local database which records data of internal and external examinations. In cases of incompleteness, the original patient health records from outpatient centers were reviewed, and the missing data was requested and included.

### Preoperative localization studies

Five different localization modalities were applied to patients in varying combinations including ultrasound of the neck, technetium-99m-sestamibi-scintigraphy, C-11-methionine-positron emission tomography computed tomography (C-11-methionine PET-CT), magnetic resonance imaging (MRI), and selective parathyroid venous sampling (SPVS). Ultrasound was usually performed by an experienced specialist in nuclear medicine, endocrinology, or an endocrine surgeon, respectively. The imaging procedures were performed at University Hospital of Wuerzburg or by one of the collaborating specialized outpatient centers. SPVS was performed by an interventional radiologist at the University Hospital of Wuerzburg (Department of Radiology).

The retrospective analysis of the imaging studies revealed that some single localization studies failed to clearly define the position of the adenoma between the upper and lower quadrants, even in cases when a re-review of the imaging was performed. In addition, intraoperative definition of upper or lower parathyroid gland can be difficult in cases of repeat surgery. Based on this, we considered localization by preoperative imaging studies as correct when the lateralization (right or left) of the adenoma was verified intraoperatively.

### Biochemical diagnostics and intraoperative PTH measurements

For all included patients, the diagnosis of pHPT was confirmed biochemically by measurements of PTH and Ca^2+^ in our laboratory which was complemented with urine calcium measurements. If previous diagnostics were inconsistent or incomplete, remaining parameters were amended during the standard diagnostic routine before surgery.

Intraoperatively, quick intact parathormone monitoring was performed 15 min after removal of the suspicious parathyroid gland. An adequate drop of intact parathormone was assumed by a significant drop of PTH to normal values in the range of 10% of the preoperative levels according to the Rome criteria [15].

### Statistical analysis

Statistical analysis was performed with using IBM SPSS Version 25. GraphPad Prism 7.0 and Microsoft Office Excel 2010 were used for graphical presentation and additional analysis. Categorical variables were calculated with the chi^2^ test. *p* values <0.05 were considered statistically significant.

## Results

### Patient characteristics

Among 643 patients who underwent surgery due to pHPT at the University Hospital Wuerzburg from 2006 to 2020, 28 of 30 patients were eligible for our analysis according to the inclusion criteria. One patient was excluded because of a recurrent/persistent pHPT due to MEN1, which required complete parathyroid resection without extended localization diagnostics after focused removal of one parathyroid gland in the primary operation. Another patient suffered from known parathyreomatosis; therefore, localization diagnostic was expected to provide no meaningful results.

All patients had significant hypercalcemia and elevated PTH levels (Table 1). In eleven patients, thyroid surgery was the primary operation and pHPT occurred later, whereas all other patients showed persistent or recurrent pHPT. Three patients had a combination of thyroid and parathyroid surgery in their history together in one intervention. The presence or persistence of symptoms including organ involvement of the kidney, musculoskeletal system, gastrointestinal tract, or psychic symptoms led to the indication for repeat surgery in all of these patients. Median age at the timepoint of repeat surgery was 53.3 years with a range from 33 to 75 years (male patients 47.1 years, female patients 57.35 years). In our cohort, 27 patients were diagnosed with a single adenoma, while only one patient suffered from multiglandular disease.

**Table 1:** Patient characteristics

				Preoperative parameters				Intraoperative characteristics				Postoperative parameters				
	Patient sex	Age, y*	Previous surgery	Recurrent or persistent pHPT	PTH	Ca^2+^	Vocal cord mobility	Localization adenoma	Bilateral vs. focused surgery	Operation time (min)	PTH decline**	Pathological confirmation	Size of the adenoma (cm)	Biochemical cure	PTH	Ca
1	m	33	TS, PS	Persistent	139	3.0	n.i.	l (intrathyroid)	F	54	Y	Y	0.2	Y	< 3.0	2.3
2	f	66	TS	-	182	2.8	n.i.	r u	F	184	Y	Y	2.6	Y	26.6	2.5
3	f	75	TS	-	109	2.7	n.i.	l lo	BE	120	Y	Y	2.8 x 1.5	Y	13.0	2.2
4	f	66	PS	Persistent	381	2.7	n.i.	l lo	F	210	Y	Y	1.5 x 1.3	Y	46.6	2.4
5	f	75	TS, PS	Persistent	257	2.9	n.i.	l u	F	120	Y	Y	2.0 x 0.7	Y	< 3.0	2.5
6	f	36	PS	Persistent	245	2.8	n.i.	r io	BE	95	N (245/45)	Y	3.5 x 1.5	Y	4.9	2.5
7	f	74	TS, PS	Persistent	143	2.8	n.i.	l u	F	169	Y	Y	1.3	Y	< 3.0	2.7
8	m	50	PS	Persistent	296	2.6	n.i.	l u	F	161	Y	Y	1.5 x 1.8	Y	12.5	2.6
9	f	56	TS	-	90	2.8	n.i.	not identified	BE	149	N	N	-	N	49.4	2.5
10	m	61	PS	Recurrent	144	2.8	N/A	r (autotransplant)	F	14	Y	Y	1.2 x 0.9	Y	14.0	2.5
11	f	63	TS	-	45	3.1	n.i.	l lo	F	105	Y	Y	1.5	Y	12.3	2.3
12	f	53	TS	-	200	2.7	n.i.	l u	BE	131	Y	Y	2.4	Y	16.8	2.4
13	m	44	PS	Recurrent	184	2.3	n.i.	l lo	F	103	N (184/42)	Y	0.3	Y	25.5	2.1
14	m	33	PS	Persistent	160	2.6	n.i.	l u	F	112	N (160/37)	Y	1.5	Y	37.0	2.4
15	m	33	PS	Persistent	98	2.6	n.i.	l lo + r u + r lo	BE	147	Y	Y	0.6, 0.3, 0.3	Y	< 3.0	2.0
16	f	34	PS	Recurrent	218	2.9	n.i.	r u	F	54	Y	Y	2.1	Y	< 5.0	2.5
17	f	38	PS	Recurrent	75.7	2.8	n.i.	r lo	F	129	Y	Y	0.7	Y	8.5	2.2
18	f	62	PS	Recurrent	96.6	2.7	n.i.	r u	F	63	Y	Y	1.8 x 1.2	Y	23.4	2.5
19	m	42	PS	Recurrent	84	2.7	n.i.	r u	BE	281	N (84/52)	Y	1.7	Y	28.1	2.6
20	m	50	PS	Recurrent	319	2.7	n.i.	r lo	BE	69	Y	Y	1.9	Y	12.6	2.5
21	f	58	TS	-	70	2.8	n.i.	r u	F	152	Y	Y	1.5	Y	< 3.0	2.4
22	m	41	PS	Recurrent	229	2.7	n.i.	l u	F	159	Y	Carcinoma	3.5 x 1.7	Y	< 3.0	2.3
23	m	68	TS	-	295	2.8	n.i.	l lo	F	69	Y	Y	1.5	Y	< 3.0	2.7
24	m	63	TS	-	204	2.8	n.i.	l lo	F	104	Y	Y	2.5 x 1.5	Y	20.0	2.5
25	f	24	PS	Persistent	162	3.0	n.i.	not identified	BE	339	N	N	-	N	71.8	2.5
26	f	63	TS	-	221	2.7	n.i.	l lo	F	169	N (371/57)	Y	1.6 x 0.9	Y	57.0	2.4
27	f	69	TS	-	259	2.8	n.i.	r lo	F	151	Y	Y	3.2 x 1.5	Y	< 5.0	2.7
28	f	63	TS	-	81	2.6	n.i.	r lo	F	159	Y	Y	0.6 x 1.2	Y	<5.0	2.2

*n*=28

*Age at timepoint of repeat surgery

*f* female, *m* male, *PTH* parathormone (normal range 12–65ng/l), *Ca*^*2+*^ calcium (normal range 2.0–2.7mmol/l), *TS* thyroid surgery, *PS* parathyroid surgery, *n.i.* no impairment, *r* right, *l* left, *u* upper, *lo* lower, *N* no, *Y* yes, *F* focused surgery (only one side), *BE* bilateral exploration

**An adequate PTH decline in intraoperative quick PTH monitoring was defined as drop of PTH below 10% of the basic value or 5ng/l

### Diagnostic workup of all patients

On average, 2.71 (range 1–5) diagnostic procedures were applied to each patient leading to a detection of an adenoma in 25/28 patients (89.3%). Nearly all patients received ultrasound and sestamibi scintigraphy as a first approach to localize the parathyroid adenoma. Ultrasound was carried out in 26 patients (92.9%) and led to the detection of a suspicious structure in only 14 patients (53.9%). This localization was later confirmed intraoperatively in 13 patients (92.9%). Among the 26 patients having sestamibi scintigraphy, 17 patients (65.4%) showed a focal enhancement suggesting the presence of a parathyroid adenoma. This localization was later confirmed intraoperatively in all cases (100%). C-11-methionine PET-CT was a less commonly used diagnostic tool and performed in 10 patients (35.7%). A suspicious structure was detected which was later confirmed to be a parathyroid adenoma intraoperatively in all but one patient (90.0%). MRI studies were applied to 5 patients (17.9%). The detection of an adenoma was possible in one case (20.0%) and confirmed intraoperatively, whereas no detection of a parathyroid adenoma by MRI was possible in all other cases. Selective parathyroid hormone venous sampling (SPVS) was conducted in 10 patients (35.7%). In 9 patients (90%), a site of PTH excess was identified, whereas in one case, no clear site of PTH excess was detected. In 8 out of 9 (89%) cases in which PTH excess could be attributed to a specific site by SPVS, the presence of the parathyroid adenoma was confirmed intraoperatively (Table 2). To get a better impression of the diagnostic workup, it was analyzed separately with respect to the initially performed surgery.

**Table 2:**
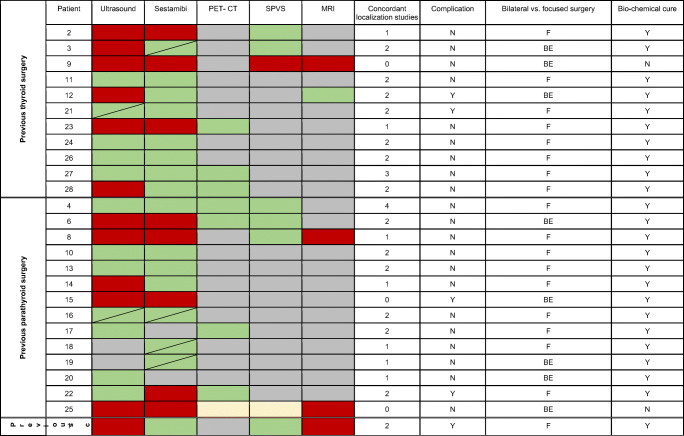
Localization modalities of patients with previous thyroid surgery, parathyroid surgery, and combined thyroid and parathyroid surgery (combined surgery) and correlation with outcome

*Sestamibi* technetium-99m-sestamibi-scintigraphy, *PET-CT* C-11-methionine-PET-CT, *SPVS* selective parathyroid hormone venous sampling, *MRI* magnet resonance imaging; a correct localization is defined as identical lateralization in localization study and surgery; *N* no, *Y* yes, *F* focused surgery (only one side), *BE* bilateral exploration

### Diagnostic workup in patients with thyroid surgery as primary operation

To detect parathyroid adenoma, ultrasound and sestamibi scintigraphy were carried in all 11 patients that underwent thyroid surgery as a primary operation. For 4 patients, ultrasound and sestamibi scintigraphy provided a clear result. One patient received the first ultrasound at an outpatient center and no adenoma was detected. Therefore, C-11-methionine PET-CT was performed. A second ultrasound in our clinic then detected a suspicious lesion. The remaining 6 patients received either C-11-methionine PET-CT or selective venous parathormone sampling or MRI. In one patient, no adenoma could be detected although 4 different diagnostic procedures had been carried out. On average, 2.64 imaging modalities were performed in patients with previous thyroid surgery (Table 2; upper part).

### Diagnostic workup in patients with parathyroid surgery as a primary operation

The 3 patients who had thyroid and parathyroid surgery together as a primary surgery all received ultrasound, sestamibi scintigraphy, and selective parathyroid hormone venous sampling. Two patients were further diagnosed using C-11-methionine PET-CT and one using MRI (Table 2; lower part).

The 14 patients who had surgical treatment of pHPT as a primary surgery received a very heterogenous diagnostic workup. In 3 patients only, one method to detect parathyroid adenoma was performed. Six patients underwent two imaging modalities; in five cases, they demonstrated conclusive findings. Three different diagnostic tools were applied to one patient, four imaging modalities to three patients, and one patient received extensive localization effort when five imaging approaches were performed. Numerous different combinations of diagnostics were detected (Table 2; middle part). On average, 2.5 imaging modalities were applied to patients with foregoing parathyroid surgery.

### Specificity and sensitivity of diagnostic approaches

Finally, specificity and sensitivity of the diagnostic tools was calculated (Table 3). While neck ultrasound and sestamibi scintigraphy showed a sensitivity of 54.2% and 70.8% respectively, with a positive predictive value (PPV) of 100%, for PET-CT a sensitivity of 100% with a PPV of 90.0% was calculated. SPVS showed a sensitivity of 100% and a PPV of 83.3%. MRI diagnostic only showed a sensitivity of 33.3%. The size of the detected parathyroid glands as revealed later during surgery did not correlate with higher detection rates of parathyroid adenomas in all diagnostic approaches.

**Table 3:** Sensitivity and specificity of localization studies

	Sensitivity (%)	Specificity (%)	PPV (%)
Ultrasound	54.2	100	100
Sestamibi scintigraphy	70.8	100	100
C11-methionine-PET-CT	100	n/a	90
MRI	33.3	100	100
SPVS	100	50	83.3

*PPV* positive predictive value, *Sestamibi* technetium-99m-sestamibi-scintigraphy, *PET-CT* C11-methionin-PET-CT, *SPVS* selective parathyroid hormone venous sampling, *MRI* magnet resonance imaging; n/a no calculation possible

### Surgical approach

In total, all 28 patients received repeat surgery as therapy of pHPT. The identification of an adenoma was successful in 26 patients (92.9%). Despite extensive re-exploration, no adenoma was found in two patients (7.1%).

In 20 patients (71.4%), a unilateral, focused surgical approach was sufficient to detect and remove the adenomatous parathyroid gland. In all other 8 patients (28.6%), an exploration of several parathyroid glands or bilateral exploration was required. The average operation time for unilateral and bilateral exploration was 122.1 min (range 14 to 169 min) and 166.4 min (range 69 to 339 min), respectively.

After identification and removal of suspicious tissue, the diagnosis was confirmed histopathological as parathyroid gland with reduced fat proportion for all patients suggesting the presence of parathyroid adenoma: first, on frozen section analysis during surgery, later, in paraffin-embedded sections. In one patient, a parathyroid carcinoma was diagnosed. In 21 patients (75.0%), intraoperative quick PTH monitoring showed an adequate drop of intact parathormone defined as levels below 10% of the basic value or below 35ng/l. Five patients (17.9%) did not fulfill the criteria but showed a considerable decline of intact parathormone (range of decline: 18.5–22.8% of the basal level). In these cases, the decision to terminate surgery was made by an experienced surgeon considering preoperative localization studies, macroscopic presentation of the adenoma, histopathologic features in fresh frozen sections during surgery, and extent of PTH decline. The two patients without intraoperative localization of the adenoma showed no changes in parathormone levels during surgery. For the one patient suffering from multiglandular disease, preoperative imaging did not reveal localization of a suspicious gland. Therefore, the patient underwent bilateral exploration. For all glands, frozen section analysis was performed. In 3 of four glands, adenomatous transformation was detected, and the glands were removed. Calcium and PTH were normalized postoperatively and the patient was cured.

Biochemical cure was defined as normalization of calcium and PTH levels in the postoperative assessment 24–48h after surgery and was achieved in 26 out of 28 patients (92.9%) after surgical intervention. As expected for the two patients in whom no adenoma was detected, no biochemical cure was evident.

### Complications

In this study, the typical procedure-related complications of bleeding and recurrent laryngeal nerve palsy were analyzed for each patient. In addition, hypocalcemia and paresthesia were not considered as complications but documented as adverse and unavoidable events. In 28 surgical interventions, six events were detected. None of the patients experienced a postoperative bleeding complication. No patient showed postoperative biochemical hypocalcemia. Five patients (17.9%) developed temporary paresthesia like numbness and tingling of the hands, feet, and face. No patient mentioned muscle cramps. All symptoms abated by the timepoint of discharge from the hospital (3–6 days). Recurrent laryngeal nerve palsy is one of the most significant complications in repeat neck surgery of the parathyroid gland. No patient showed symptoms of nerve palsy like hoarseness or altered voice quality. Twenty-seven out of 28 patients received an otorhinolaryngological control of vocal cord movement. One patient refused the postoperative examination. Although free of symptoms and good intraoperative neuromonitoring signals, one patient (3.6%) showed a paresis of the right vocal cord in the postoperative control. She was closely monitored, and vocal cord movement started to recover 2 months after surgery and then recovered completely. Therefore, no long-term complication was detected in this cohort of patients.

### Outcome related to preoperative imaging

**Fig. 1:**
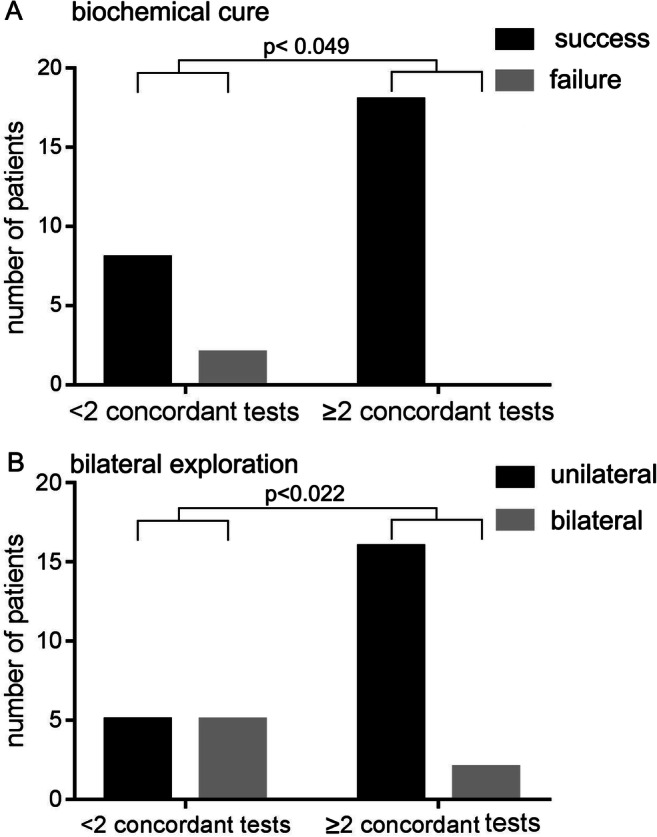
Cure rate and operative aspects with regard to concordant preoperative diagnostics. A The number of patients with biochemical cure is shown for the group with <2 or ≥2 concordant imaging. B The number of patients that required bilateral exploration is shown for the group with <2 or ≥2 concordant imaging

Outcome was compared to the extent of preoperative diagnostics: In patients with 2 or more concordant imaging modalities, all patients (18/18) were biochemically cured, whereas failure of biochemical cure occurred in 20% of patients (2/10) with less than 2 concordant imaging modalities (*p*< 0.049) (Fig. 1A). The number of patients requiring bilateral exploration was significantly reduced (*p*< 0.022) in the group that had two or more concordant imaging modalities to 11.1% (2/18) compared to the group with less than two concordant imaging modalities (50%; 5/10) (Fig. 1B). Since only one patient had transient recurrent nerve palsy, no significant differences in complications between the two groups were detected. The rate of patients with transient paresthesia (*n*=5) was not different between the groups.

## Discussion

Repeated neck surgery can be demanding due to scar formation and anatomical distortion. The overall morbidity in repeated thyroid/parathyroid surgery has declined due to treatment in specialized centers. However, there remains a significant overall morbidity including an increased risk for permanent nerve palsy, permanent hypoparathyroidism, and the failure to cure patients.

The present study adds new insight from a cohort of patients undergoing repeated neck surgery due to pHPT. We show that biochemical cure was achieved in 92.5% of patients. Apart from MRI, all diagnostic modalities, including neck ultrasound, sestamibi scintigraphy, and C-11-methionine PET-CT, were suitable for the localization of parathyroid adenoma before repeated surgery with a high positive predictive value. In addition, selective parathyroid hormone venous sampling was effective tool regionalize or lateralize the parathyroid adenoma. Two concordant preoperative diagnostic modalities increased the rate of biochemical cure and significantly reduced the risk of bilateral neck exploration. The complication rate with one transient recurrent nerve palsy was low. Both, previous thyroid and parathyroid surgery showed similar rates of complications and biochemical cure making the type of primary surgery irrelevant.

### How much imaging is required for successful repeat surgery?

Despite a large body of literature and recommendations in the context of repeated surgery in patients with persistent/recurrent pHPT or foregoing thyroid surgery, it remains almost impossible to answer the question how much imaging diagnostic is sufficient or required for a successful repeated procedure. The American Association of Endocrine Surgeons suggests non-invasive imaging studies and, if unsuccessful, complementary invasive imaging [16]. However, it does not account for false positives when one imaging modality is used, which may mislead the surgeon and even trigger an adverse outcome. Henry recommended that localization studies with at least two concordant results should be performed before re-operation [17], although supporting evidence was weak. Our current data with a small but robust cohort of patients assessed with timely diagnostic tools support this recommendation.

### Which imaging modalities are reasonable before repeated surgery in a first step?

Neck ultrasound and sestamibi scintigraphy are the most frequently used and established methods for the localization of pathological parathyroid glands. Ultrasound is the most economic at 50€, while costs for sestamibi scintigraphy and SPVS range from 750€ to 1.500€ and appear to be largely comparable to PET-CT [18]. Costs will vary considerably depending on the health care system in which these procedures are performed. Aside from the expenses of preoperative imaging, it must be considered that long-term costs after failed repeat surgery may be even higher, which warrants the best preoperative diagnostics in these special cases.

Sensitivity for neck ultrasound after previous parathyroid or thyroid surgery varies from 60.5 to 74%, and the positive predictive value (PPV) was calculated to be 67–93% [2, 8, 13]. In our study, the sensitivity of neck ultrasound was 56.5% with a positive predictive value (PPV) of 100%. Here, sestamibi scintigraphy localized adenomas with a sensitivity of 69.6% and a PPV of 100%. For initial localization before primary surgery, a sensitivity of 79–100% and PPV of 77–88% have been described [2, 8, 13]. Also, the accuracy of technetium-99m-sestamibi-scintigraphy before re-operative parathyroidectomy is reduced compared to the situation before primary surgery [19]. The radiation dose of 0.0085 mSv applied to the patient during sestamibi scintigraphy is considerably lower compared to the radiation dose during PET-CT (7-10 mSv) and SPVS. According to our own data, radiation dose is in the range of 150 mSv for SPVS depending on interindividual properties of the patients.

This supports previous suggestions that ultrasound and sestamibi scintigraphy should be carried out as a first diagnostic step before repeat surgery in cases of recurrent and persistent pHPT. However, concordant localization of the adenoma was possible in only 10 of 21 patients that received ultrasound and sestamibi scintigraphy. In this case, extended diagnostics should be carried out.

### What are the options for extended imaging diagnostics?

C-11-methionine PET-CT has previously been shown to be a reliable method with a sensitivity of 79–100% [20] especially in cases with atypical localization [21–23]. After previous neck surgery, C-11-methionine PET-CT shows comparably good results with a sensitivity of 79–94% and a PPV of 87–94% [13, 22]. Our results showed a sensitivity of 100% with a PPV of 88.9%.

Recently, F-18-choline PET-CT was reported to detect parathyroid adenoma with higher accuracy than C-11-methionine PET-CT making it a promising alternative [24].

The use of MRI shows variable results where the overall sensitivity was 64% before primary surgery but only 47% with repeat surgery and showed the lowest sensitivity of all diagnostic tools [8, 25]. This is confirmed in our cohort of patients as we found a sensitivity of only 33.3% for MRI diagnostics. Therefore, we do not recommend MRI as a sufficient tool for the localization of adenomas in recurrent pHPT.

### Is hormone venous sampling an appropriate approach to detect parathyroid adenoma in patients planned for repeat surgery?

In cases of failure or discordant results, selective parathyroid hormone venous sampling (SPVS) is a method with the potential to regionalize or lateralize a parathyroid adenoma without giving the exact position and relation to other structures. The value of SPVS in patients with previous surgery was questioned since venous drainage might be changed due to scar formation and ligation of vessels during the first surgery. In our cohort of patients, we found a sensitivity of 100% and a PPV of 83.3% for SPVS. These findings are supported by previous data where a sensitivity of 83.3–100% in recurrent or persistent pHPT with a PPV of 77–100% was reported [2, 8, 13, 26, 27] [28].

In our cohort, no patient experienced complications after SPVS which is line with previous reports [26, 29]. However, possible complications such as vessel dissection, bleeding, hematoma, or anaphylaxis caused by contrast media are serious, and the exposure to a radiation dose of up to 150 mSv is higher compared to that of a PET-CT. Considering these factors, we recommend performing SPVS as a final step in the preoperative diagnostic workup.

## Conclusion

This study gives insight into the preoperative imaging modalities before repeat surgery in patients with pHPT and supplements previous findings regarding effective preoperative localization diagnostics and patient outcome. The limitations of this study are its retrospective design, the low number of patients, and heterogeneous cohort undergoing repeat surgery for recurrent/persistent pHPT or following thyroid surgery.

**Fig. 2:**
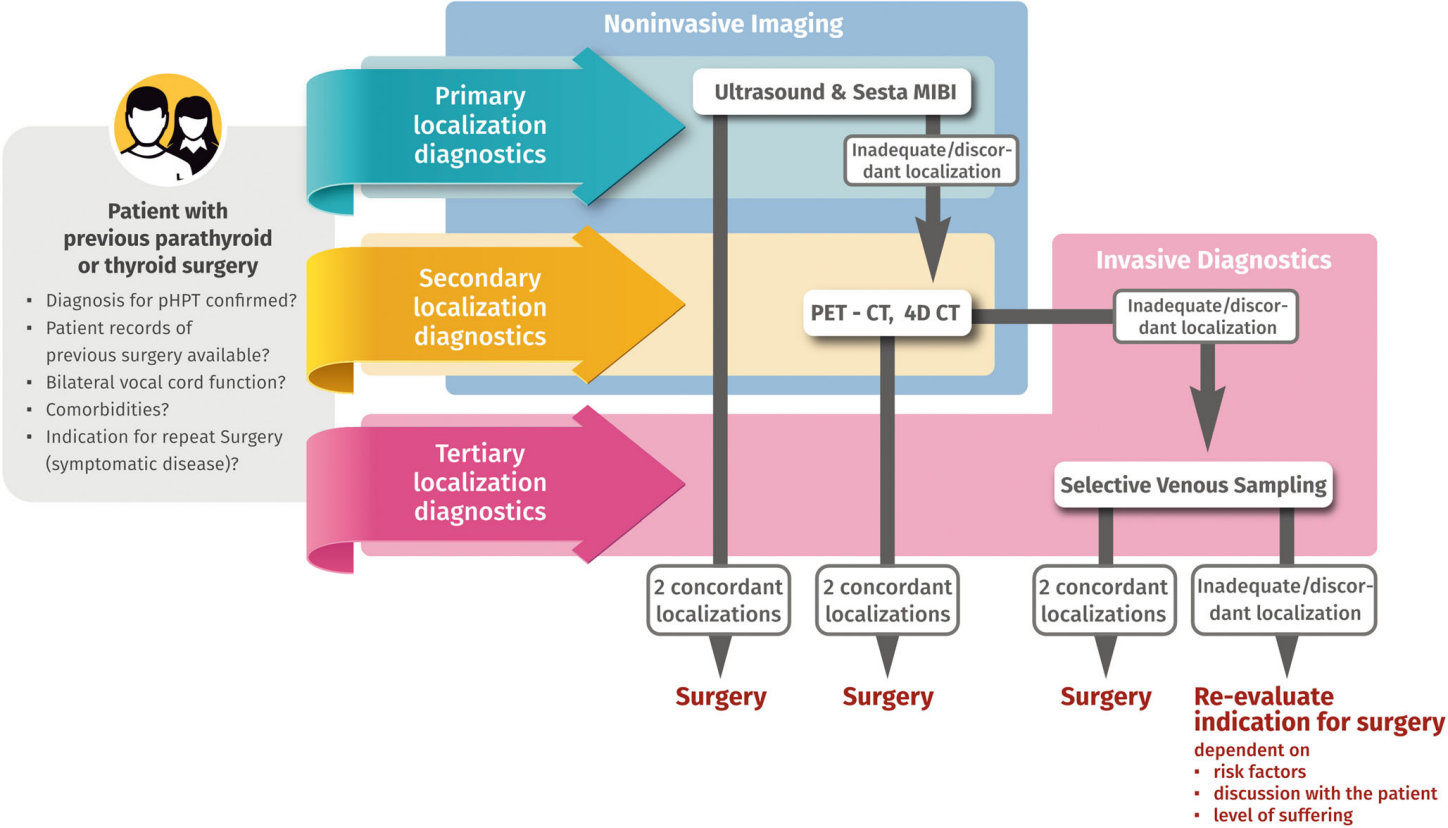
Algorithm for preoperative imaging before repeat surgery. For details see text

Based on our data and the current literature [2], we present an algorithm for imaging before repeat surgery in cases of recurrent or persistent pHPT or after previous thyroid surgery (Fig. 2). We recommend performing neck ultrasound and sestamibi scintigraphy as a first step on every patient with upcoming repeat surgery. In cases of insufficient or discordant results, we recommend performing PET-CT. In cases of insufficient noninvasive imaging, SPVS should be performed to optimize conditions for surgery in order to receive the best therapeutic results possible.

## Data Availability

All data are presented in this manuscript.
